# Supplementary material for: Learning curve and surgical outcome of robotic assisted colorectal surgery with ERAS program
Source: Sci Rep. 2022 Nov 29;12:20566. doi: 10.1038/s41598-022-24665-w (PMC9709162; doi:10.1038/s41598-022-24665-w)
Supplement: Supplementary file 1 — Supplementary Information 1. [file 41598_2022_24665_MOESM1_ESM.zip › LSC Raw data-Ñ[▒K/122/O_ñw╝╨░O▒Kñσ.pdf]

正式摘要(電子簽章：000000212481066)

請選擇...reporter

臺中榮民總醫院

Operation Note

[redacted]

- 手術日期：2021/03/18
- 手術醫師：[redacted]，CR [redacted]，Fellow [redacted]，R [redacted]
- Anesthesia : GE
- Position : Supine
- Aseptic agents : Betadine Aqua , Zephnan

Diagnosis

- Pre-operative diagnosis : Malignant tumor of hepatic flexure
- Post-operative diagnosis : s/p laparoscopic right hemicolectomy

Surgical Indication

- For removing the primary tumor

Operation

- 1. Laparoscopic right hemicolectomy with side to side anastomosis (OpenGIA 75-1.5 X2 were applied)
  2. Ileo-colic artery, right colic artery and right branch of middle colic artery were all ligated via Hemo-clips.
  3. Take down of hepatic flexure : (+)
  4. Cut margin: proximal (ileum) 8 cm, (colon) >15 cm, distal 5 cm.
  5. Repair of mesentery defect : (+). Done via continuous running suture with 3-O mersilk.
  6. No drainage tube was placed.
  7. ADHESION BARRIER FILM was placed below the central abdominal wound.

Finding

- 1. A 3 X 3 ulcerative tumor located at hepatic flexure site.
  2. No other gross tumor was found.
  3. There was no visible liver metastasis or visible peritoneal seeding.
  4. Multiple palpable LNs were noted over ileocolic vessel area.

Procedure

- 1. General endotracheal anesthesia was induced and then the patient was placed in the supine position.
  2. Preoperative antibiotics were given. A Foley catheter and nasogastric tube were placed. The abdomen was prepped and draped in standard sterile fashion.
  3. A small vertical incision was made left to the umbilicus and a 12mm trocar was inserted into the peritoneal cavity by open method.
  4. Pneumoperitoneum was established with CO2 inflation. Laparoscope was inserted and then a 12mm trocar were inserted over the central wound of abdomen.
  5. Another 5mm trocar was inserted over LLQ of abdomen ; The other 5mm trochars were inserted over RUQ of abdomen and RLQ of abdomen, respectively. A 12mm trochar was inserted over LUQ of abdomen.
  6. The small bowel was inspected and retracted to downward by grasper. The peritoneum overlying the mesentery was then scored with electrocautery and the ileocolic artery was identified, ligated with endoclips, and transected via Harmonic scalpel.
  7. The remaining vessels to the cecum were also divided and ligated. Using electrocautery and Harmonic scalpel, omentum was freed from the transverse colon and lesser sac was entered.

- 8. The right branch of middle colic, right branch artery and right colic artery were all further ligated via Hemo-clips and Harmonic scalpel.
  9. After the vessel were all under controlled, the colon was freed from its peritoneal attachments along the line of Toldt from the cecum to the hepatic flexure.
  10. Hemostasis was checked in the operative field. Cecum, ascending colon and hepatic flexure were mobilized and then laparoscope was removed.
  11. The midline incision was extended over left peri-umbilical area.
  12. The ascending colon was delivered into the incision and points of transection were selected proximally and distally.
  13. Side to side anastomosis was performed with two OpenGIA 75-1.5 mm. Interrupted 3-0 silk Lambert sutures were used to re-inforcement at outer layer. Mesentery defect was repaired via continuous running suture with 3-O mersilk.
  14. The anastomosis was checked and found to be intact and widely patent.
  15. The small bowel and colon was put back into the abdomen. Pneumoperitoneum was established again.
  16. Possible bleeder was examed again. The orientation of the small bowel was checked thoroughly.
  17. ADHESION BARRIER FILM was placed below the central abdominal wound.
  18. The fascia was closed with interrupted sutures via Num.1 Vicryl. The fascia of the 12mm trochar wound was closed with 1-0 Vicryl.
  19. The wound was further closed with 3-O Vicryl, and the skin was applied with SurgiSeal. Finally covered with Medipore.
  20. The patient tolerated the procedure well and was brought to the POR in stable condition.
- Estimated blood loss: minimum
- 器械數量: 正確
- 紗布數量: 正確

Image

- PAINT
  - abdomen

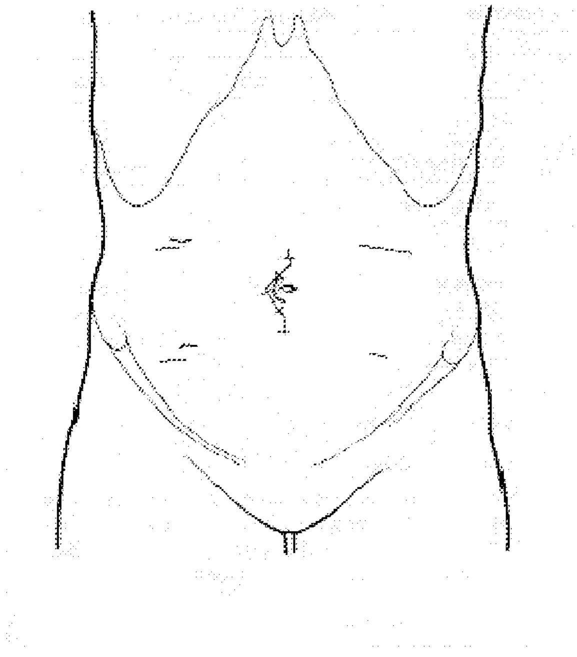

  - colon & rectum

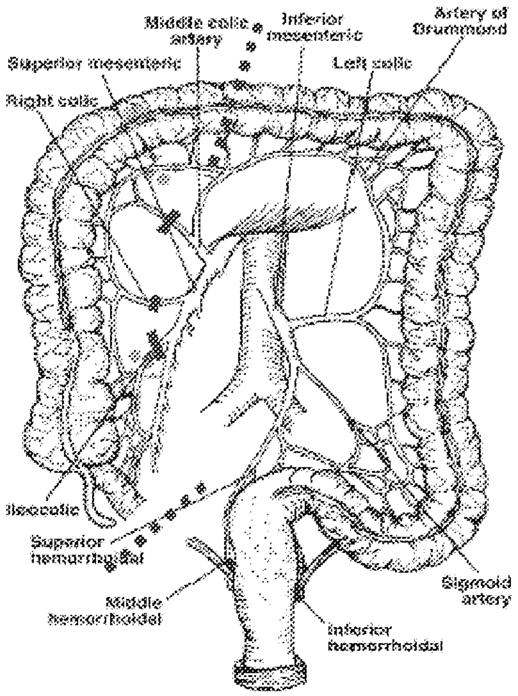

Blue dot : suspicious LNs

- The resected A-colon and T-colon

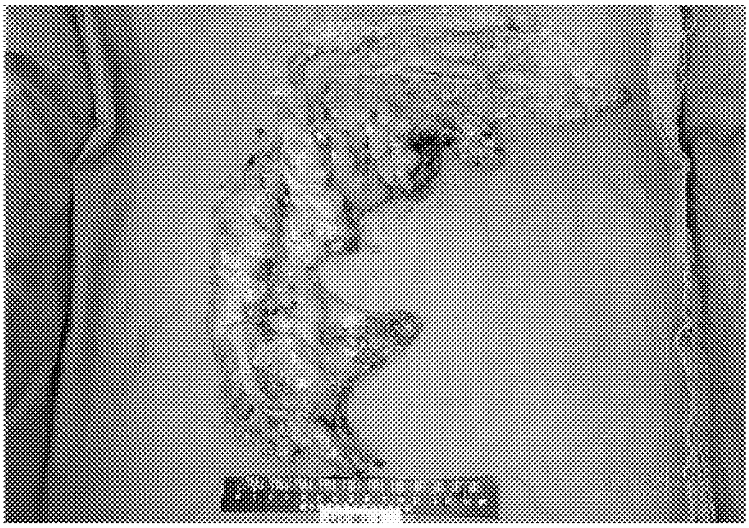

The tumor could be clearly seen

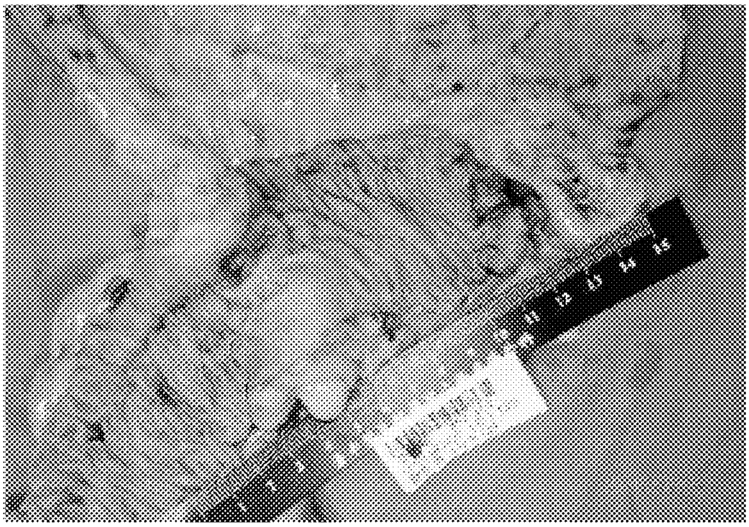

主任 2062F蔣鋒帆 / 醫師 2301H林陳彥

正式摘要(電子簽章：**000000212481066)**

請選擇...reporter

doctorRank:B
